# 

**Published:** 1912-05-04

**Authors:** 


The following appointments have been made at the Dreadnought Hospital, Greenwich: Dr. Henry MacCormac, M.B., M.R.C.P., to be assistant physician for diseases of the skin; Dr. R. S. Lawson, M.B., Ch.B., and Dr. B. Phillips, M.B., Ch.B., to be house physicians; and Dr. C. Ede, M.B., B.C., and Dr. A. Gibson, M.B., Ch.B., to be house surgeons.

### OUR BUREAU OF INFORMATION.

### A New Home of Rest for Gentlewomen.

There are none more difficult to help, none who need help more, than working women of education and gentle nurture who fall on evil days through ill-health. The days have long gone by when it was considered an indignity for gentlewomen to earn their own living. In families of small means the daughters and sisters no longer sit idly at home crushed by a feeling of dependence and conscious of adding to the burdens of those they love. But it remains true to-day, as in the past, that the struggle to be independent is doubly hard on women who have lived a sheltered life until the wage-earning period began. Such women, however courageous and indomitable under misfortune, are peculiarly exposed to suffering when through ill-health their salary comes to an end and the means of keeping up the home is taken from them. How very often it befalls our sisters in every profession that rest and freedom from worry is recommended in vain to ward off some illness or to complete recovery. And what an ironical prescription this sensible piece of advice must often sound! We see the girls and women all round us—in the train, in the church, in the street—whom we would fain lift up on a magic carpet and waft away to fresh air and ease until the strained, wretched look vanishes from the face and faith and health are restored. And yet it is not so easy as it might seem to get to know of those who would really benefit most from such a period of recreation. There is a section of ladies living on small means who are always, it is true, very thankful to profit by enterprises founded to assist women of their special class. It is in no ungentle spirit that we would intimate that their claims should give way before those who are dependent on their own exertions to maintain their independence. Among the great army of educated women working for their living as clerks, governesses, social workers, or in any other of the countless occupations now open to women, there are innumerable instances to be found of candidates for the kind of help we have indicated.

Will our readers bring to our notice any ladies in need of rest and kindly care in good air and surroundings? Through the great generosity of a lady who is founding a new home with this aim in view we are enabled to pass on such cases to her consideration. The home will be opened about the middle of May in a favourite seaside locality, and the foundress writes: "We are most anxious to help ladies who have to earn their own living and are in need of a change and rest, or anyone who has undergone an operation recently." No case of an infectious nature is admissible, nor can consumptive cases be received, as this would not be fair to the other guests, who will share a general sitting-room and dining-room. Nor can ladies who require special care and nursing be received. Those who work for others, as so many of our readers are privileged to do, are most likely to encounter the weary and overdone for whom this delightful holiday home has been arranged. A small sum weekly is payable by those who are in a position to make some contribution towards their maintenance.

---

### NEEDS AND HELPERS.

### Inexpensive Homes Wanted.

"C. F. S."—For a lady who has been treated at Duxhurst, but has now overcome the tendency to alcoholism [103A].

"Francis."—For girl of 14, who has developed somewhat suddenly a bad type of neurasthenia, taking the form of religious delusions and melancholia. Fairly strong in health, affectionate, very conscientious, and anxious to overcome morbid feelings. South of England desirable though not essential [104A].

### Employment for Dull Girl.

Place wanted for a strong girl of 19, not very bright, but able and willing to do housework or laundrywork. Suitable for small institution to scrub or wash, or for small family under mistress willing to supervise. No wages need be given, only necessary clothing and a little pocket-money. Prepaid answers to "Wantage" forwarded.

---

F. H. Heath.—The London address of the National Association for the Prevention of Consumption is 20 Hanover Square, W.

"Francis."—It is very cheering to know that you value The Hospital so much and are interested specially in this section of it. Perhaps you will act as our correspondent in your island in connection with the "Brotherhood of Service." Please let us know if this is so.

Prepaid answers from Approved Homes, addressed to "Francis" or to "C. F. S.," under cover to The Editor of The Hospital Bureau of Information, 28 Southampton Street, Strand, London, will be forwarded.

---

### Rules for Correspondents.

Everyone who uses or wishes to help our Bureau of Information must be kind enough to observe the following rules:—

(1) Postal replies cannot be given. Exception will only be made in very special cases at the Editor's discretion.

(2) Applications referring to non-dependants where the need relates to business or employment must be accompanied by a postal order for 5s., when the requirement shall have publicity in these columns.

(3) All applicants for insertion in our list of Approved Homes must send a registration fee of 5s. The omission of this leads to delay and gives avoidable trouble.

(4) *All communications* for this department *must be accompanied by the coupon* to be found at the bottom of the third page of the cover on the inside, and should be addressed to the Editor of the Bureau of Information, c/o The Hospital, 29 Southampton Street, Strand, London, W.C.

---

Readers of The Hospital are invited to bring these columns under the notice of their friends who are engaged in philanthropic work of any description. We want the co-operation of district nurses, district visitors, and health visitors. We want the clergy to send us on particulars of cases which need institution treatment. All who are trying to help their neighbours are faced with the problem of fitting the remedy to the need. It is this problem we aim at solving.

### Berkel's Patent Slicing Machine.

Prominent among the list of "Up-to-date Kitchen Specialities" last week there should have appeared a detailed account of Berkel and Parnall's slicing machine. Not merely as a labour-saving device has it recommended itself to hospital managers, but for its cleanliness in slicing, and the fact that waste is avoided in slicing meats, whether hot or cold, bread, and other commodities to fourteen different thicknesses. A patent guard, moreover, protects the operator—a fact of importance to employers since the Workmen's Compensation Act. The "Berkel" is made in three sizes, and can of course be fitted with a friction clutch and pulley for power driving if desired. Any shaped piece of meat can be placed upon the machine, which has become a regular part of kitchen apparatus in most of the large institutional kitchens throughout the country. It should be added that the Incorporated Institute of Hygiene has awarded its certificate to the manufacturers, whose works and chief offices are at 6 Bow Common Lane, London, E.

### THE EDITOR'S LETTER-BOX.

### HOSPITAL CONSTRUCTION COMPETITIONS.

### EAST SUSSEX HOSPITAL.

*To the Editor of* The Hospital.

### Appointments.

Mr. W. McAdam Eccles, F.R.C.S. Eng., has been elected surgeon to St. Bartholomew's Hospital.

Mr. Francis A. Duffield, M.B., Ch.B. Edin., has been appointed demonstrator in experimental physiology and pharmacology at the University of Sheffield.

Dr. Herbert French, M.A., M.D. Oxon, F.R.C.P. Lond., assistant physician to Guy's Hospital, etc., has been appointed honorary consulting physician to the Surrey Dispensary, Great Dover Street, S.E., in succession to the late Sir Samuel Wilks, Bart., M.D., F.R.S.

The following appointments, etc., have been made at the West Bromwich District Hospital: Mr. Priestley Smith, M.B., F.R.C.S., of Birmingham, to be honorary consulting ophthalmic surgeon, in place of Mr. A. E. Chesshire, resigned. The office of honorary anæsthetist has also been created, and Mr. W. J. McCardie, M.B., of Birmingham, has been appointed to it.

Mr. R. C. Harkness has been appointed second house surgeon to the Leicester Infirmary. He graduated with first-class honours at Edinburgh University in 1909, passed M.R.C.S., L.R.C.P. in 1911, and took the degree of F.R.C.S. (Eng.) in 1911. Mr. Harkness has held senior resident posts at the Edinburgh and Manchester Royal Infirmaries, Wolverhampton General Hospital, and London Temperance Hospital. Mr. H. W. Ingham, M.B., Ch.B. (Leeds), has been apoointed assistant house surgeon to the same institution.

